# Detection value of free cancer cells in peritoneal washing in gastric cancer: a systematic review and meta-analysis

**DOI:** 10.6061/clinics/2016(12)10

**Published:** 2016-12

**Authors:** Francisco Tustumi, Wanderley Marques Bernardo, Andre Roncon Dias, Marcus Fernando Kodama Pertille Ramos, Ivan Cecconello, Bruno Zilberstein, Ulysses Ribeiro-Júnior

**Affiliations:** Hospital das Clínicas da Faculdade de Medicina da Universidade de São Paulo, São Paulo/SP, Brazil

**Keywords:** Gastric Carcinoma, Peritoneal Washing, Peritoneal Lavage, Cytology, Carcinoembryonic Antigen, RT-PCR

## Abstract

Intraperitoneal free cancer cells in gastric adenocarcinoma are associated with a poor outcome. However, the true prognostic value of intraperitoneal free cancer cells is still unclear, leading to a lack of consensus in the management of gastric cancer. The aim of the present study is to perform a systematic review and meta-analysis to analyze intraperitoneal free cancer cells-positive patients with regard to tumor oncologic stage, recurrence, grade of cellular differentiation, and survival rates and to analyze the clinical significance of intraperitoneal free cancer cells with regard to prognosis. Databases were searched up to January 2016 for prognostic factors associated with intraperitoneal free cancer cells, including oncologic stage, depth of neoplasm invasion, lymph nodal spread, differentiation grade of the tumor, and recurrence and survival rates. A total of 100 studies were identified. Meta-analysis revealed a clear association between intraperitoneal free cancer cells and a poor prognosis. intraperitoneal free cancer cells -positive patients had higher rates of nodal spread (risk difference: 0.29; *p*<0.01), serosal invasion (risk difference: 0.43; *p*<0.01), recurrence (after 60 months of follow-up, risk difference: 0.44; *p*<0.01), and mortality (after 60 months of follow-up, risk difference: 0.34; *p*<0.01). Intraperitoneal free cancer cells are associated with a poor outcome in gastric cancer. This surrogate biomarker should be used to guide therapy both prior to and after surgery.

## INTRODUCTION

Peritoneal dissemination is the most common pattern of recurrence in gastric cancer, even after a potentially curative resection. This characteristic may be attributable to possible intraperitoneal dissemination of malignant cells already present at the time of surgery or to surgical manipulations. Current knowledge on intraperitoneal free cancer cell (IFCC) positivity in gastric cancer demonstrates that these cells are associated with a poor prognosis and advanced oncologic stages. Additionally, high recurrence rates, mainly due to peritoneal dissemination, and poor median survival are associated with cytology detection 1-3.

Based on these data, the Japanese Classification of Gastric Carcinoma: 3^rd^ English Edition 4 and the 7^th^ Edition of the AJCC Cancer Staging Manual: Stomach 5 consider conventional cytology positivity in peritoneal fluid to be an indicator of stage IV disease.

Several institutional protocols are used to manage IFCC-positive patients, including chemotherapy, prompt gastrectomy, neoadjuvant treatment, peritoneal infusion, hyperthermic peritoneal chemotherapy, or palliation alone. However, none of these techniques are accepted worldwide as a gold standard therapy.

The investigation of peritoneal washing for IFCCs in gastric cancer patients remains controversial. Little is known about the actual burden of IFCC positivity and its accuracy for predicting an outcome. Moreover, a lack of consensus exists in its routine practice, methods of detection 6, and association with clinical pathological variables.

Thus, the aim of this study was to perform a systematic review and meta-analysis, investigating patients positive for IFCCs detected via different methods, regarding the neoplasm oncologic stage, recurrence rates, grade of cellular differentiation, and survival rates and to analyze the clinical significance of IFCCs with regard to prognosis.

## METHODS

The construction and modeling of the present study were guided by the Preferred Reporting Items for Systematic Reviews and Meta-Analyses (PRISMA) statement 7.

### Database search

A literature search was performed in MEDLINE using the following search terms: (((“Stomach Neoplasms/cytology”[Mesh]) AND ((Peritoneum OR Peritoneal OR abdominal cavity OR ascitic fluid OR washing OR lavage)))) OR (((cytology AND gastric cancer)) AND ((Peritoneum OR Peritoneal OR abdominal cavity OR ascitic fluid))). Other databases searched included LILACS, CENTRAL, Cochrane, CINAHL, and Scopus as well as grey literature.

No attempts were made to locate unpublished material.

### Inclusion criteria

- Patients with confirmed gastric adenocarcinoma submitted to preoperative peritoneal washing/lavage evaluation (open, laparoscopic, or by paracentesis) for IFCCs (conventional cytology with Papanicolaou, Giemsa, or Hematoxylin-eosin staining); molecular methods, such as RT-PCR for carcinoembryonic antigen (CEA), cytokeratin (CK20), and melanoma-associated gene (MAGE); and immunohistochemistry.
- Studies that evaluated the prognosis (i.e., oncologic stage, survival, recurrence rate, or grade of cellular differentiation).
- Prospective or retrospective studies.
- Studies selected by both of two reviewers.

### Exclusion criteria

- Data could not be extracted from pooled results.
- Patients submitted to a neoadjuvant approach prior to the peritoneal washing/lavage procedure.
- Presence of other primary malignancy.
- Case series, case reports, animal models, conference proceedings, editorials, and letters.
- Review articles and meta-analyses were excluded from meta-analysis.
- Studies with no full-text.

### Idiom

No restriction.

### Search period

No restriction. The search was performed up to January 2016.

### Outcomes

- Recurrence rate
- Recurrence site: lymph node, peritoneal, or other organs (local recurrence or hematogenous spread)
- Mortality
- Oncologic stage
- Serosal invasion
- Lymph node spread
- Grade of cellular differentiation

### Statistical analysis

Absolute numbers for the outcome parameters were extracted and analyzed with Review Manager Version 5.3 software (Copenhagen: The Nordic Cochrane Centre; The Cochrane Collaboration, 2014).

We performed subgroup analysis and sensitivity tests to explore the causes of statistical heterogeneity in which the effect of single studies on the heterogeneity value was tested. Forest plots were used for graphical exploration of heterogeneity. A funnel plot was used to identify publication bias.

## RESULTS

### Studies characteristics

Of the selected articles, 20 were excluded because they lacked the information necessary for meta-analysis, such as serosal invasion, oncologic stage, neoplasm dissemination, and grade of cellular differentiation. In total, 100 (1-3, 8-104) eligible trials were identified and reviewed, and 91 were included in the meta-analysis. Cumulatively, 16,913 gastric cancer patients were evaluated. In 41 studies analyzed, all patients were submitted to curative intention surgery.

We assessed the quality of the studies using the Newcastle-Ottawa Scale (NOS). In terms of study quality, the cohort studies were considered to be of fair (scores of 4–6) to good (scores of 7–9) quality, but two articles were considered low quality (scores of 1-3).

Of the 100 eligible trials, data describing conventional cytology were available for 68 papers; data regarding PCR-CEA were available for 27; and data regarding PCR-CK20 were available for 5. Other studies also evaluated Ber-Ep4, MAGE, RT-LAMP, or a combination of techniques used to detect IFCCs.

Most studies performed peritoneal washing/lavage similarly to the method described by Nakajima et al. 69. The peritoneal cavity was washed with 50 to 200 ml of normal saline. After stirring, the fluid was collected. Thirty-three studies collected fluid from the Douglas space, 16 collected fluids from the Douglas and left subphrenic spaces, and 5 collected fluid from the perigastric surroundings. The remaining studies collected fluid from different combinations of recesses. Peritoneal washing/lavage was performed by laparotomy in 81%, by laparoscopy in 15.2%, and by drainage tube in 3.8% of the studies.

Data were collected from 16 countries. The median follow-up across all studies was 36 months (range 12-108 months).

The prevalence of IFCCs ranged from 2 to 72%, with a median of 27%. Considering only conventional cytology studies, the median prevalence was 19.3% (range 2-61%). Considering only PCR-CEA, the median prevalence was 27.8% (range 15-63%). Considering only PCR-CK20, the median prevalence was 27.9% (range 15-39%).

### IFCC and oncologic stage

The present study analyzed the oncologic stage according to the UICC/AJCC system 6^th^ edition 105. For this purpose, IFCC detection alone was not considered stage IV.

The pooled data of the network meta-analysis showed that IFCC detection was associated with a significantly higher risk of stage III or IV compared with stage I or II (risk difference: 0.41; 95% CI: 0.33–0.49; n=4,258 patients; I^2^=88%, *p*<0.00001) (see Figure 1). The sensitivity analysis failed to identify outliers. A random-effects analysis method was used to adjust for inter-study heterogeneity.

For the subgroup analysis, conventional cytology studies 1-3,12,16-19,24,49,54,79,94,96 (risk difference: 0.34; 95% CI: 0.2–0.48; n=2,373 patients; I^2^=93%; *p*<0.00001) and PCR-CEA 31,36,38,49,77,93,94,97,104 (risk difference: 0.5; 95% CI: 0.36–0.63; n=1,073 patients; I^2^=83%; *p*<0.00001) were reviewed by comparing stage III or IV patients with stage I or II patients.

Comparable results were identified (risk difference: 0.32; 95% CI: 0.19–0.44; n=600 patients; I^2^=55%; *p*<0.00001) when analyzing studies that evaluated oncologic stages according to the UICC/AJCC system 7^th^ edition 26,31,38,59,97.

### IFCC and serosal invasion

The pooled data of the network meta-analysis showed that IFCC detection was associated with a significantly higher risk of serosal invasion than tumors that did not invade the serosa (risk difference: 0.43; 95% CI: 0.38–0.48; n=11,511 patients; I^2^=89%, *p*<0.00001) (see Figure 2). The sensitivity analysis failed to identify outliers. A random-effects analysis method was used to adjust for inter-study heterogeneity.

For the subgroup analysis, conventional cytology studies 2,3,10,11,15,16,18,19,22,25,29,34,40,41,43,46,48-57,60,63,69,70,78,81,83,87,92,94,96,99,101 (risk difference: 0.39; 95% CI: 0.35–0.43; n=2,374 patients; I^2^=93%; *p*<0.00001) and PCR-CEA 28,36-38,48-51,57,58,64,70,76,77,83,89,93,94,97,101, (risk difference: 0.51; 95% CI: 0.45–0.57; n=2,612 patients; I^2^=66%; *p*<0.00001) were reviewed.

### IFCC and lymph node spread

The pooled data of the network meta-analysis showed that IFCC detection was associated with a significantly increased risk of lymph node spread compared to cancer with no lymph node involvement (risk difference: 0.29; 95% CI: 0.23–0.34; n=7,718 patients; I^2^=89%, *p*<0.00001) (see Figure 3). The sensitivity analysis failed to identify outliers. A random-effects analysis method was used to adjust for inter-study heterogeneity.

For the subgroup analysis, conventional cytology studies 1-3,12,14,16,18,19,25,29,41,43,46,51,52,54-57,61,63,78,81,92,94,96, (risk difference: 0.25; 95% CI: 0.18–0.31; n=5,008 patients; I^2^=87%; *p*<0.00001) and PCR-CEA 31,36,38,48,57,58,64,76,77,93,94,97,101,104 (risk difference: 0.3; 95% CI: 0.15–0.45; n=1,464 patients; I^2^=93%; *p*<0.00001) were reviewed.

### IFCC and grade of cellular differentiation

The pooled data of the network meta-analysis showed that IFCC detection was associated with a significantly increased probability of having poorly differentiated tumors compared to well or moderately differentiated tumors (risk difference: 0.15; 95% CI: 0.12–0.17; n=7,232; I^2^=65%, *p*<0.00001).

A sensitivity analysis was performed by repeating the network analysis after omitting 3 studies with a high risk of bias 31,102,103. The final result revealed a risk difference of 0.15 (95% CI: 0.13–0.18; n=6,784; I^2^=43%, *p*<0.00001) (see Figure 4).

For the subgroup analysis, conventional cytology studies 2,10,11,18,19,34,40,41,43,55-57,69,78,81,87,89,92,94-96,102, (risk difference after excluding 2 outliers 102,103: 0.17; 95% CI: 0.14–0.2; n=5,437 patients; I^2^=39%; *p*<0.00001) and PCR-CEA 31,57,64,77,93,94,97 (risk difference: 0.08; 95% CI: 0.01–0.15; n=805 patients; I^2^=55%; *p*<0.04) were reviewed.

### IFCC and recurrence

The recurrence rate was assessed for gastric cancers treated with curative intention surgery.

The pooled data of the network meta-analysis showed that IFCC detection was associated with a significantly increased risk of recurrence. For recurrence after 24 months of follow-up 15,16,28,72, the risk difference was 0.38 (95% CI: 0.25–0.51; n=360 patients; I^2^=57%, *p*<0.00001). For recurrence after 60 months, the risk difference was 0.44 (95% CI: 0.32–0.56; n=2,176 patients; I^2^=88%, *p*<0.00001) (see Figure 5).

For IFCC-positive patients, the mean recurrence rate was 55.35% after 24 months and 68.73% after 60 months. For IFCC-negative patients, the mean recurrence rate was 16.77% after 24 months and 31.36% after 60 months.

### IFCC and sites of recurrence

For gastric cancers treated with curative intent surgery, studies were assessed regarding peritoneal recurrence, lymph nodal recurrence, or recurrence in other organs.

For peritoneal recurrence, the presence of IFCCs predicted a risk difference of 0.48 (95% CI: 0.38–0.59; n=2,683 patients; I^2^=86%, *p*<0.00001) (see Figure 6). The sensitivity analysis failed to identify outliers. A random-effects analysis method was used to adjust for inter-study heterogeneity.

For lymph nodal recurrence, the presence of IFCCs predicted a risk difference of 0.05 (95% CI: 0.00–0.1; n=1,553 patients; I^2^=29%, *p*=0.05) (see Figure 7).

For local or hematogenous recurrence, the presence of IFCCs did not predict a poor prognosis (risk difference: 0.02; 95% CI: -0.03, 0.07; n=1,355 patients; I^2^=23%, *p*=0.22) (see Figure 8).

### IFCC and mortality

The pooled data of the network meta-analysis showed that IFCC detection was associated with a significantly increased risk of mortality.

For mortality after 12 months of follow-up 9,13,15,20,56,57,63,85,92, the risk difference was 0.26 (95% CI: 0.19–0.33; n=1,765 patients; I^2^=48%, *p*<0.00001). One study was omitted after sensitivity analysis 92.

For mortality after 24 months 9,13,20,29,61,63,84,92, the risk difference was 0.4 (95% CI: 0.33–0.48; n=934 patients; I^2^=35%, *p*<0.00001). One study was omitted after sensitivity analysis 9.

For mortality after 60 months, the risk difference was 0.34 (95% CI: 0.29–0.38; n=1,811 patients; I^2^=50%, *p*<0.00001). Two studies were omitted after sensitivity analysis 69,72 (see Figure 9).

For IFCC-positive patients, the mean mortality rate was 43.5% after 12 months, 75% after 24 months, and 72.3% for studies that analyzed mortality after 60 months. For IFCC-negative patients, the mean mortality rate was 16.6% after 12 months, 43.2% after 24 months, and 41.2% after 60 months.

For the subgroup analysis, studies that exclusively evaluated patients who submitted to curative intention surgery were assessed.

For mortality after 12 months, the risk difference was 0.35 (95% CI: 0.24–0.45; n=799 patients; I^2^=13%, *p*<0.00001). One study was omitted after sensitivity analysis 92.

For mortality after 24 months, the risk difference was 0.34 (95% CI: 0.24–0.44; n=717 patients; I^2^=35%, *p*<0.00001). One study was omitted after sensitivity analysis 9.

For mortality after 60 months, the risk difference was 0.42 (95% CI: 0.37–0.47; n=995 patients; I^2^=58%, *p*<0.00001). One study was omitted after sensitivity analysis 18.

## DISCUSSION

The present study evaluated the burden of IFCC positivity in gastric cancer by analyzing the individual data of each included study. The strengths of our study include the following: the study strategy was designed to be comprehensive; the inclusion and exclusion criteria and data extraction were determined to reduce bias; no idiom restrictions allowed the avoidance of cultural and racial bias; and this is the one of the first studies to analyze the true burden effect of IFCC positivity on recurrence rates (and sites of recurrence) and mortality rates. A limitation of this study was that some of the comparisons had a high level of heterogeneity.

Pecqueux et al. 106 also analyzed the relationship between IFCCs and survival and recurrence rates. However, they compared studies that evaluated recurrence and survival rates at different times, which compromised the validity of the findings.

Therefore, the present study assessed survival and recurrence rates at 1-, 2-, and 5-year follow-up evaluations. IFCC was associated with higher early and late mortality. Additionally, our study evaluated sites of recurrence and showed that recurrence was mainly due to peritoneal dissemination.

Similar to Pecqueux et al. 106, our study also revealed a high level of heterogeneity for recurrence rates. This finding could be explained by the different follow-up programs of each oncologic center, including different adjuvant therapies and methodologies for diagnosing recurrence.

To explore the causes of statistical heterogeneity, we performed subgroup and sensitivity analyses in which the effects of single studies on the heterogeneity value were tested. A funnel plot was used to identify publication bias. If publication bias was identified, the study was excluded from the analysis.

IFCC was associated with lymphatic spread (risk difference: 0.29; 95% CI: 0.23–0.34, *p*<0.00001) and lymph nodal recurrence (risk difference 0.05; 95% CI: 0.00–0.1, *p*<0.05). This result may be explained by confounding variables (IFCC actually could be associated with neoplasm depth, which would subsequently be associated with lymph nodal spread and recurrence). None of the trials explored these data, and future studies could investigate a possible link between lymphatic and peritoneal dissemination, which has been suggested by some authors 107.

IFCCs are also associated with locally advanced tumors, especially those with serosal invasion, but can also be found in earlier clinical stages of gastric cancer. Part of the mechanism by which advanced tumors disseminate into the peritoneum is likely associated with the area of serosal invasion 53,54,79, which could contribute to tumoral cell exfoliation and seeding into the peritoneal surface 108.

When assessing for subgroup analysis, different methods of detecting IFCC presented similar results for the prognosis. Both conventional cytology and PCR-CEA were associated with advanced stage cases, serosal invasion, nodal spread, and poorly differentiated neoplasms.

Most IFCC-positive patients who were treated with curative intent surgery likely experienced minimal to no benefit from the surgery, with most experiencing early recurrence (55.35% in 24 months). The survival rate of IFCC-positive patients was 25% after 2 years.

Accordingly, preoperative peritoneal washing/lavage in gastric cancer should be strongly advised for high surgical risk patients (e.g., the elderly, low status performance patients, and patients with incapacitating comorbidities). If IFCC positivity is determined, palliative therapy may be considered.

In low surgical risk and oncologic low risk (no serosa invasion, no lymph nodal spread, moderate or well differentiated neoplasm) patients, immediate surgery should be performed, and intraoperative peritoneal washing/lavage should be added. If IFCC positivity is determined, postoperative chemotherapy could be indicated. Clinical trials of hyperthermic intraperitoneal chemotherapy may be proposed.

The pooled data demonstrate that IFCC findings are an independent prognostic factor in gastric cancer. From this work, it can be concluded that the prognosis in surgically treated patients with gastric carcinoma is significantly affected by the presence of IFCCs at the time of gastrectomy and should guide gastric cancer management.

## AUTHOR CONTRIBUTIONS

Tustumi F was responsible for the elaboration of the project and manuscript writing. Bernardo WM was responsible for the statistical analysis. Dias AR and Ramos MF helped with manuscript revision. Cecconello I and Zilberstein B selected the articles. Ribeiro-Junior U was responsible for the elaboration of the project.

## Figures and Tables

**Figure:**
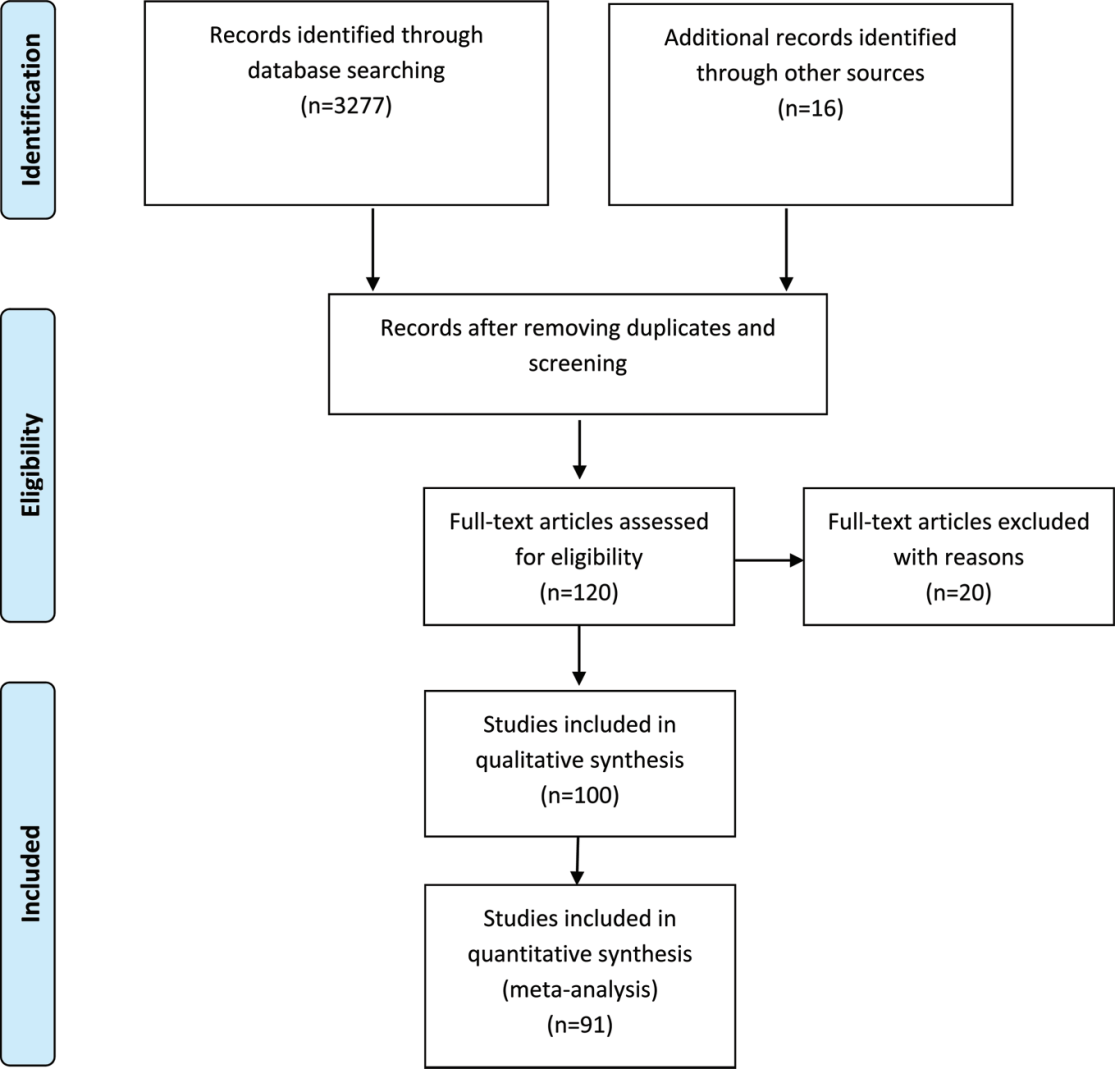


**Figure 1:**
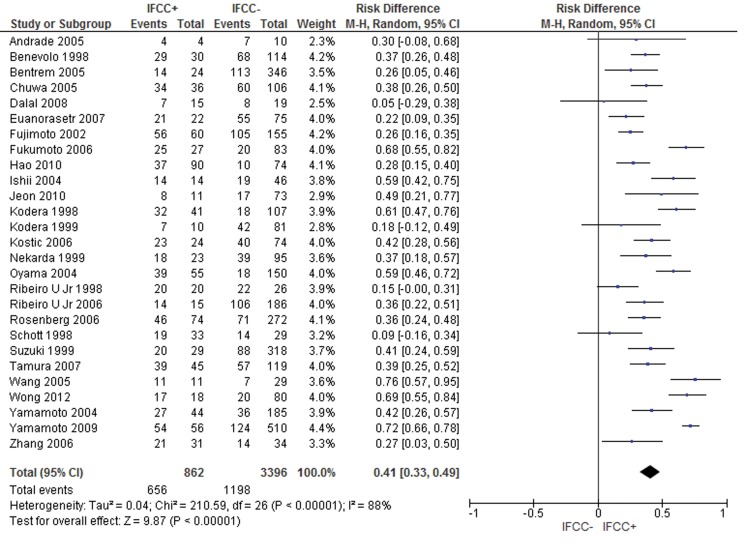
Oncologic stage according to the AJCC 6^th^ edition. A strong association was observed between IFCC detection and stages III and IV.

**Figure 2:**
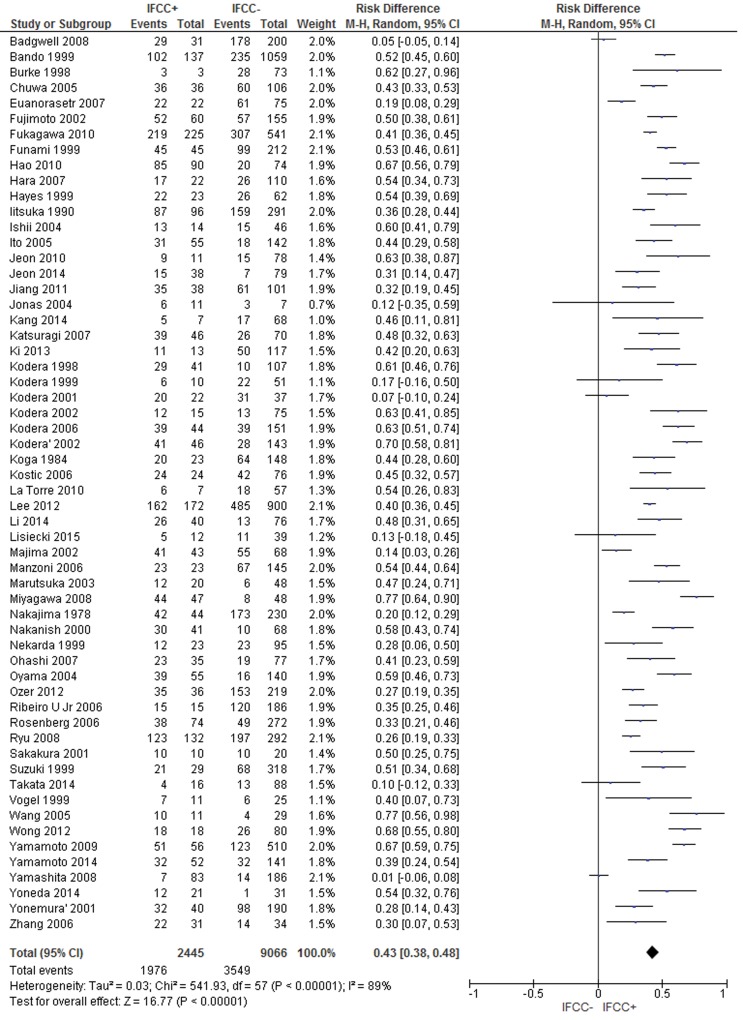
Evaluation of serosal invasion. An association between IFCC detection and serosal invasion was demonstrated.

**Figure 3:**
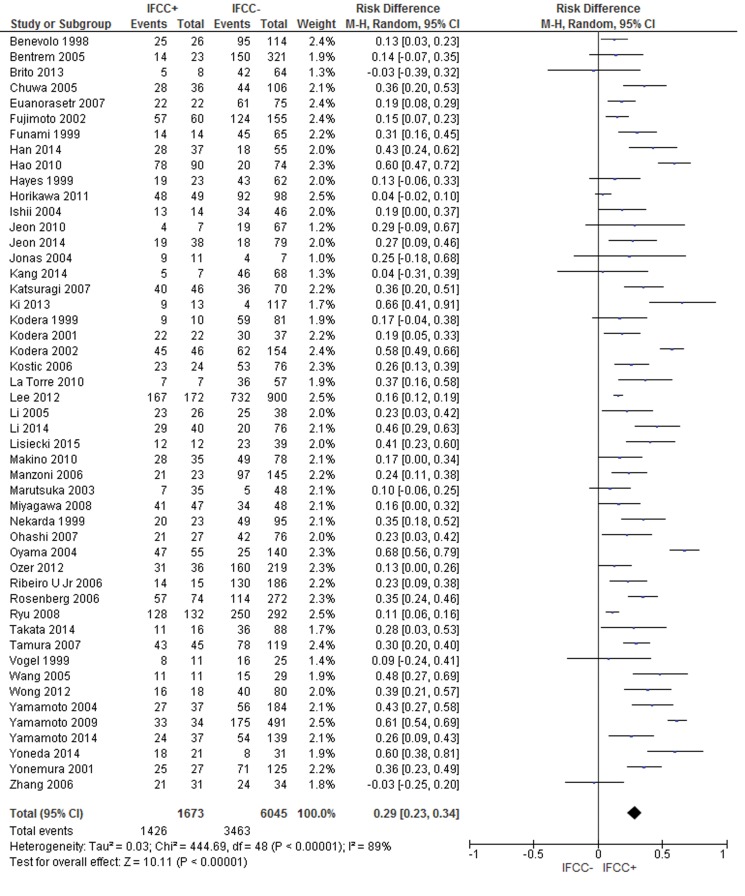
Evaluation of lymph node dissemination. A clear association between IFCC detection and lymph node metastasis was noted.

**Figure 4:**
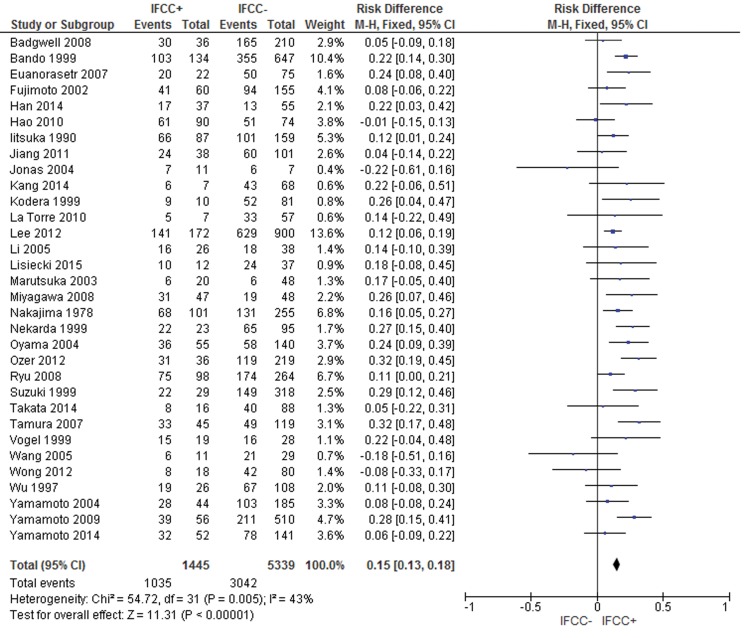
Grade of cellular differentiation. A meta-analysis revealed the prognostic value of the grade of cellular differentiation. IFCC detection exhibited a stronger association with poorly differentiated tumors than with well or moderately differentiated tumors.

**Figure 5:**
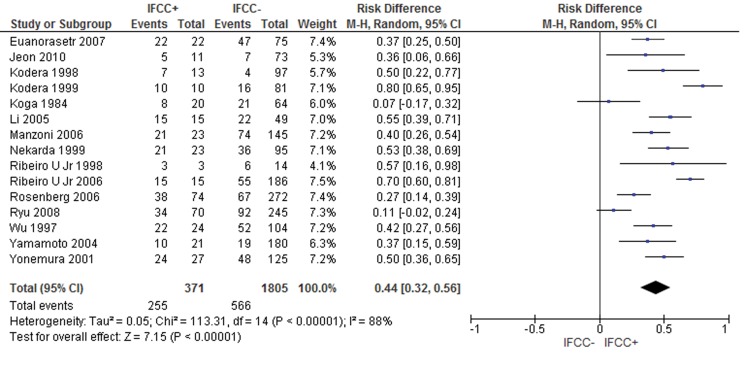
Recurrence rate. IFCCs were associated with a higher probability of recurrence after 60 months of follow-up.

**Figure 6:**
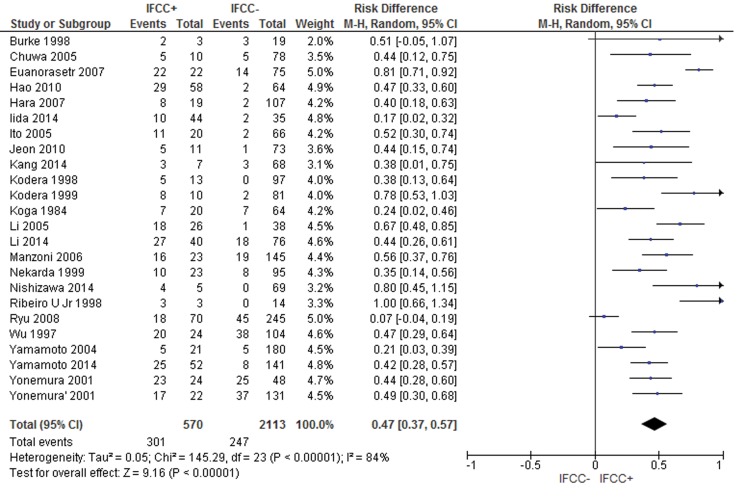
A strong association between IFCC detection and peritoneal recurrence was noted.

**Figure 7:**
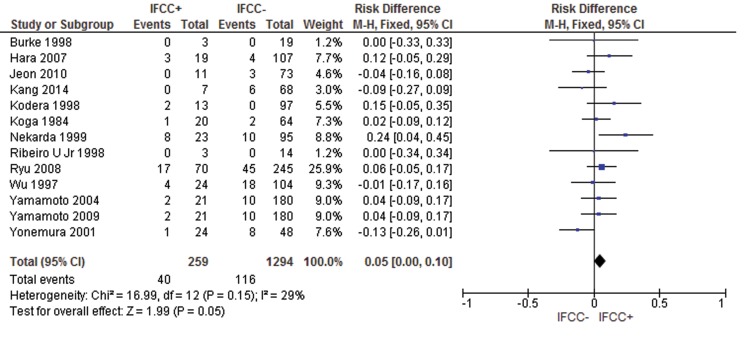
IFCC detection can also predict a higher probability of lymph nodal recurrence.

**Figure 8:**
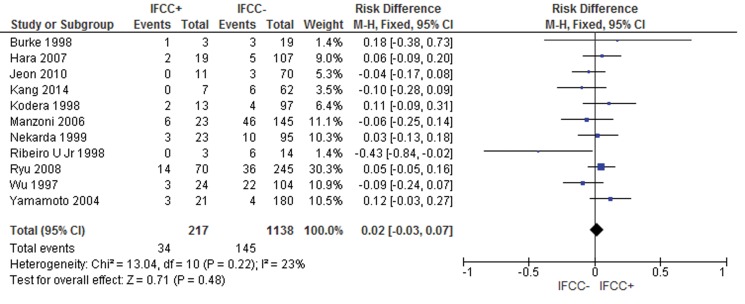
Hematogenous or local recurrence was not associated with IFCC detection.

**Figure 9:**
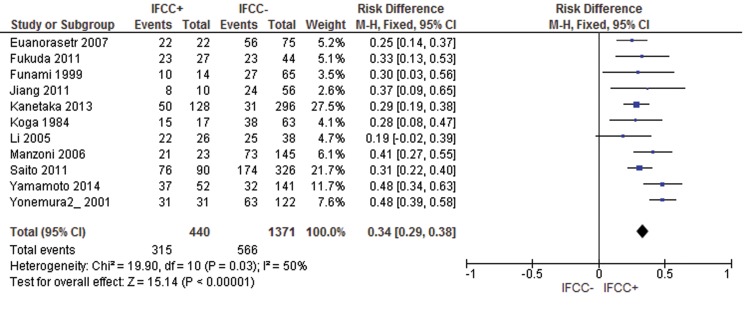
Mortality rate. IFCC detection was associated with a higher mortality rate after 60 months of follow-up.
